# Knee Angle and Stride Length in Association with Ball Speed in Youth Baseball Pitchers

**DOI:** 10.3390/sports6020051

**Published:** 2018-05-29

**Authors:** Bart van Trigt, Wouter Schallig, Erik van der Graaff, Marco J. M. Hoozemans, Dirkjan Veeger

**Affiliations:** 1 Department of Human Movement Sciences, Faculty of Behavioral and Movement Sciences, Vrije Universiteit Amsterdam, Amsterdam Movement Sciences, 1081 BT Amsterdam, The Netherlands; bartvantrigt@live.nl (B.v.T.); w.schallig@hotmail.com (W.S.); m.j.m.hoozemans@vu.nl (M.J.M.H.); h.e.j.veeger@vu.nl (D.V.); 2 Department of Biomechanical Engineering, Delft University of Technology, 2828 CE Delft, The Netherlands

**Keywords:** kinematics, biomechanics, sports, fastball

## Abstract

The purpose of this study was to determine whether stride length and knee angle of the leading leg at foot contact, at the instant of maximal external rotation of the shoulder, and at ball release are associated with ball speed in elite youth baseball pitchers. In this study, fifty-two elite youth baseball pitchers (mean age 15.2 SD (standard deviation) 1.7 years) pitched ten fastballs. Data were collected with three high-speed video cameras at a frequency of 240 Hz. Stride length and knee angle of the leading leg were calculated at foot contact, maximal external rotation, and ball release. The associations between these kinematic variables and ball speed were separately determined using generalized estimating equations. Stride length as percentage of body height and knee angle at foot contact were not significantly associated with ball speed. However, knee angles at maximal external rotation and ball release were significantly associated with ball speed. Ball speed increased by 0.45 m/s (1 mph) with an increase in knee extension of 18 degrees at maximal external rotation and 19.5 degrees at ball release. In conclusion, more knee extension of the leading leg at maximal external rotation and ball release is associated with higher ball speeds in elite youth baseball pitchers.

## 1. Introduction

The throwing technique of a baseball fastball pitch can be described as a coordinated sequence of body movements and muscular forces with the ultimate goal of arriving at the highest ball speed possible at ball release [1]. It is believed that the interaction of body segments transfers energy in a sequential pattern from the ground up to move the upper extremity joints into the right position to finally result in a high ball speed [2,3]. The lower extremities and the trunk are the main force generators during initiation of the throw [4]. For an optimal use of the trunk in force generation, the lower extremities have to be a stable base for the initiation of the rotations of the trunk and upper extremities throughout key phases of the baseball pitching action [5].

When the leading leg is extended it is braced to enhance the ability of the trunk to rotate both forward and in the axial direction simultaneously [6,7,8]. This braced leading leg was found to be associated with a high ball speed in pitchers [9]. A detailed analysis of lower limb mechanics was performed in a study by Milewski et al. [10], although they did not investigate the relation between lower limb mechanics and ball speed. A commonly held view is that pitchers who flex their knee after the moment of stride foot contact (FC) are not throwing to their highest potential [11]. This is supported by a study that reported pitchers who threw at a higher velocity had both a slower rate of knee flexion of the leading leg on landing and a higher rate of subsequent knee extension as compared with pitchers that throw at a lower velocity [12]. In addition to the knee angle at FC, greater knee extension of the leading leg was observed at ball release (BR) in faster throwing pitchers compared to slower throwing pitchers (32 SD 9° vs. 48 SD 14°) [6]. Also, Werner et al. [13] reported this association between the knee angle of the leading leg at BR and ball speed (β = −0.11, SD −0.029, *p* = 0.009). In the same study, however, a more flexed knee of the leading leg at FC was associated with a higher ball speed, which contrasts with most other studies. 

Another lower extremity parameter of interest in baseball pitching is stride length, which partly is dependent of body height and the build of the pitcher [14]. A pitcher with a larger stride length results in more forward displacement, which can result in a higher ball speed [15,16]. Furthermore, a larger stride length provides a greater moment arm for the trunk to rotate forward over the “locked leg”. Montgomery & Knudson [17] observed a positive linear relationship (*r* = 0.73) between stride length and ball speed in professional pitchers. However, this study was based on a small sample size and, to our knowledge, there are no other studies that relate stride length to ball speed, but a study has been conducted that showed that ball velocity does not have to be affected with a smaller stride length [18].

The studies described above indicate the importance of the lower extremities in achieving high ball speeds in baseball pitching [15,16,17]. Quantifying the associations of several lower extremity parameters at several instants in the pitching cycle might give additional insight in how the kinematics of the lower extremities contribute to ball speed. However, the existing literature that associates lower extremity parameters to ball speed involve only adult pitchers [6,9,12,13,17]. Exploring the association between lower extremity parameters and ball speed in youth baseball pitchers might provide additional information as this study population shows more variance in anthropometric characteristics (segment lengths, force capacities) as well as in throwing technique and ball speed. The current baseball literature that includes youth baseball pitchers as participants is only descriptive without any associations with ball speed [10,19]. Therefore, the purpose of the present study was to determine whether stride length and knee angle of the leading leg at foot contact, at the instant of maximal external rotation (MER) of the shoulder, and at ball release are associated with ball speed in elite youth baseball pitchers. It was hypothesized that a larger stride length, a more flexed knee at FC, and a more extended knee at MER and BR have a positive association with ball speed in elite youth baseball pitchers.

## 2. Materials and Methods

### 2.1. Participants

Data were collected from 52 baseball pitchers, at a mean age of 15.2 years (SD 1.7, range 10.4–18.5). Mean body height was 177.0 cm (SD 12.8, range 147.0–204.2) and mean body weight was 68.7 kg (SD 17.1, range 35.3–131.6). Of the 52 tested pitchers, 42 were right handed. Participants were recruited from the national youth baseball team as well as all (six) baseball academies in The Netherlands, at which the most talented baseball players of that region train. This research was conducted in accordance with the Declaration of Helsinki and the Department of Human Movement Sciences’ local ethical committee approved the measurement protocol. Both participants and their parents were informed of the procedure and study aims before the start of the measurements. Informed consent was obtained from the parents of the participants before involvement in the study.

### 2.2. Procedure

The measurements were performed at the indoor facilities of six baseball academies. After performing several anthropometric measurements, the pitchers were given an unlimited amount of time to warm up for pitching. They were instructed to prepare just as if they were going to pitch in a game. The pitchers wore sneakers, athletic shorts, and no shirt. They also wore their catching glove to mimic the game situation as much as possible. Once a participant was ready to pitch, he performed ten fastball pitches for data collection. The participant pitched towards a catcher behind the home plate at a distance in accordance to the participants’ age category (for players aged 13 years and younger the normal pitching distance (18.3 m) was changed to 16.5 m). Forty of the pitchers threw their pitches from a pitching mound. The other twelve pitchers threw from flat ground, because no indoor-mound was available during the measurements.

### 2.3. Data Acquisition

**Figure 1:**
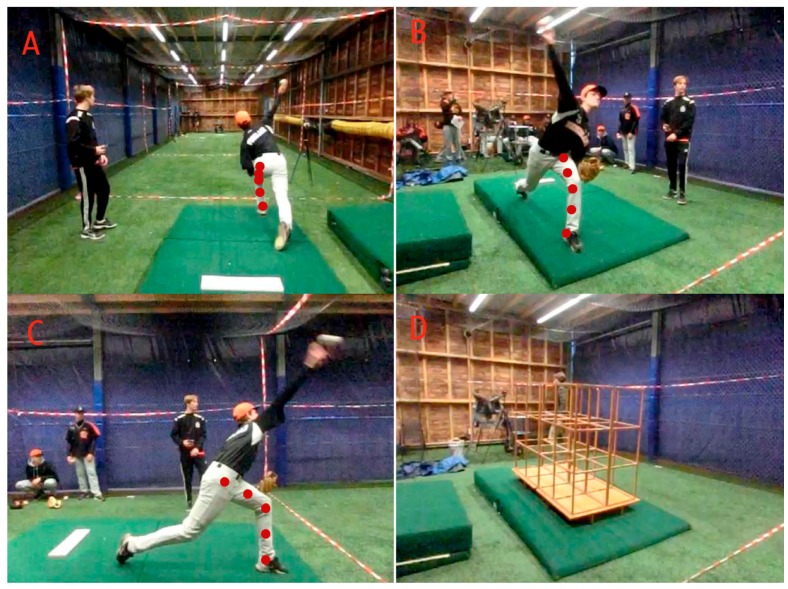
This figure shows the three different views of the three cameras for a pitch at ball release. The red circles in picture (**A**–**C**) illustrates the five marked points in that view for the direct linear transformation. Picture **D** shows one of the three pictures of the wooden reference frame which was used for calibration.

Kinematic data were collected with three high-speed video cameras (XL ZR 1000, Casio, Tokyo, Japan) at 240 Hz. The three cameras were placed approximately three meters from the pitcher to be able to film the entire pitcher from three different directions and to minimize projection errors (Figure 1). The first camera was placed behind the mound and directed towards the home plate, the second was placed sideways to the mound and the third was placed slant in front of the mound. After the setup of the cameras they were not repositioned anymore as their image was calibrated using a wooden reference frame of 1.9 × 1.35 m (visible in Figure 1D) to be able to perform 3D analyses. If one of the cameras was moved, a new calibration had to be performed. Before every pitch, a flashlight was used for synchronization of the video data of the three cameras. The ball speed (mph) reached during the pitches was measured from behind the home plate with a Stalker pro radar gun (Stalker Radar, Plano, TX, USA).

### 2.4. Data Analysis

Stride length and knee angles were calculated using MATLAB (MathWorks, Natick, MA, USA). Stride length was determined from the sagittal video, using a picture from the video at the moment of foot contact (FC). Foot contact was defined as the first moment that the heel, foot, or toe of the leading leg contacted the ground after the stride. Marking lines where taped on the ground at 1 m, 1.5 m, and 2 m, respectively from the pitching rubber. The last marking line and the pitching rubber were marked manually in MATLAB. The total amount of pixels between those markings divided by the length of 2 m, was used to obtain the conversion rate. The stride length of the youth baseball pitchers was measured and defined as the distance between the pitching rubber and the ankle joint center of the leading leg at FC. Subsequently, the relative stride length was calculated as the stride length as a percentage of the participant’s body height.

**Figure 2:**
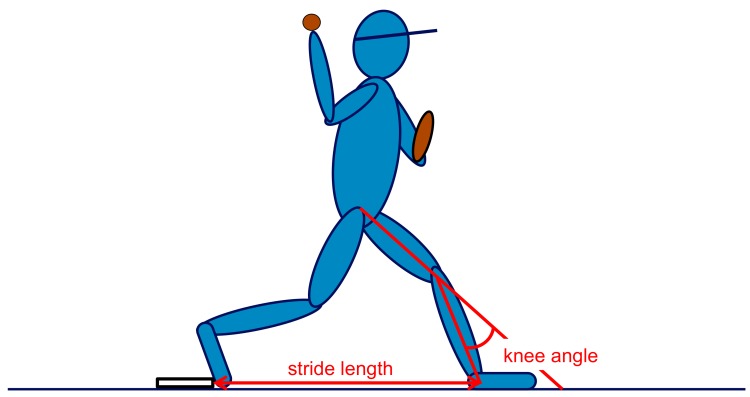
Visual explanation of the calculated stride length and knee angle calculated in 3D.

The images of the three video cameras were used to determine the 3D knee angle of the leading leg using the Direct Linear Transformation technique [20]. Synchronized pictures of all three cameras were calibrated with a wooden reference frame (visible in Figure 1D), which was designed to include as much as possible of the space in which the pitcher is moving. The synchronized pictures of the three cameras were used to determine the 3D knee angle at the time of foot contact (FC), at maximal external rotation (MER) of the shoulder and at ball release (BR). For each of the three moments in the pitch cycle (i.e., FC, MER, BR), the corresponding three pictures of the three camera positions were used to mark five points in each frame: the joint center of the ankle, the midpoint of the shank, the joint center of the knee, the midpoint of the thigh, and the joint center of the hip (see Figure 1C as an example). These points were marked manually using MATLAB, without the assistance of markers attached to the pitcher’s skin. A vector was fitted through the three points on each segment (upper leg and lower leg). Subsequently, the knee angle was calculated as the dot product of the two vectors. The knee angle was defined as the smallest angle between the vectors through the lower leg and the upper leg (Figure 2). A higher value for the knee angle thus means more knee flexion.

### 2.5. Statistical Analysis

Five of the ten pitches of each participant were included for data analysis. These five pitches consisted of the two fastest and the two slowest pitches, and one pitch that was closest to the pitcher’s average ball speed. To explore the associations between the knee angles and ball speed, and between relative stride length and ball speed, regression analysis using Generalized Estimating Equations (GEE) was used [21]. GEE are a regression analysis that considers the five selected pitches of each participant as repeated measurements and accounts for this dependency. This means that linear regression analyses could be performed without the necessity of using the average value of these five pitches. The advantage of this analysis is that within-person variation is considered when calculating the regression coefficient. An exchangeable working correlation structure was used. Relative stride length and knee angles at FC, MER, and BR (independent variables) were analyzed separately in relation to ball speed (dependent variable). In these analyses the potential confounding variables body height, age, and body weight were considered, which showed a strong correlation with ball speed (*r* > 0.8). As all potential confounding variables appeared to be highly correlated with each other (*r* > 0.75), only body height was explored for confounding the associations to prevent collinearity, except for the analysis that included relative stride length which was already expressed as a percentage of body height. Another potential confounding variable was pitching from a mound or flat ground since an independent-samples *t*-test showed that the ball speed of the mound group (30.6 m/s, SD 3.2) was significantly higher than the ball speed for the flat ground group (27.5 m/s, SD 3.4) (*t* (50) = 2.9, *p* = 0.006, Cohen’s *d* = 0.93). This variable was not correlated with body height, so collinearity was not an issue. The analysis started with a simple linear regression with one of the knee angles or the stride length as predictor variable and ball speed as outcome variable. Then the possibly confounding variables body height and mound were separately added using multiple regression analysis. If the regression coefficient of the main predictor changed more than 10% when including the potentially confounding variable, this variable was considered as a confounder [22]. The interaction between the main predictor variable and the confounding variable was also checked. However, a significant interaction was never observed, and interaction variables were, therefore, not included in the final GEE models. Standardized regression coefficients were determined as a measure of strength for the associations. All statistical analyses were performed with IBM SPSS Statistics 21.0 (IBM Corporation, Armonk, NY, USA) and a significance level of 5% was used.

## 3. Results

**Figure 3:**
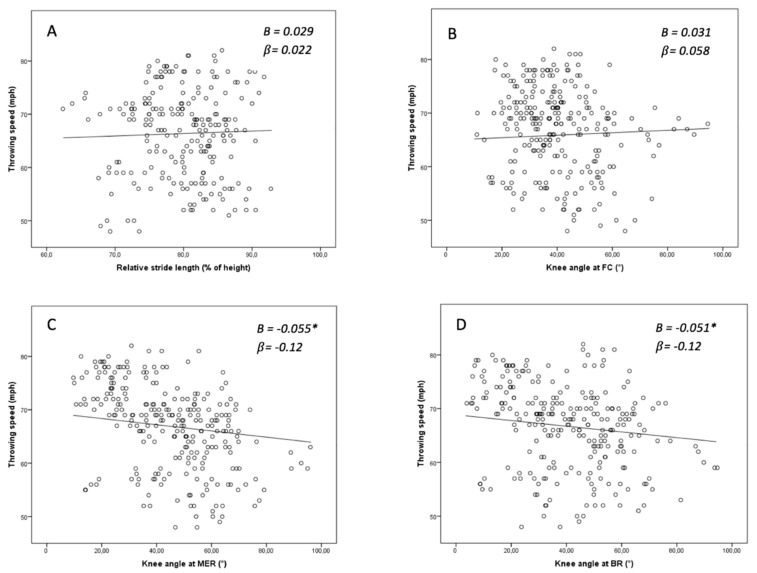
Scatterplots of observed values (including the repeated measures) of ball speed against stride length as percentage of body height (**A**), knee angle at foot contact (FC) (**B**), knee angle at maximal external rotation of the shoulder (MER) (**C**) and knee angle at ball release (BR) (**D**). Larger knee angles indicate more flexion. Regression lines are according to the adjusted regression coefficients using Generalized Estimating Equations (GEE) (see Table 1). B is the unstandardized coefficient. β is the standardized coefficient beta * *p* < 0.01.

The mean ball speed observed for all 5 pitches of all participants was 67 mph (SD 8, range 48–82). The mean stride length of the participants was 140.8 cm (SD 15.2, range 99.8–173.8) and on average 79.8% (SD 6.0, range 62.4–92.8) of their body height. Results of the multiple linear regression analysis did not show a significant association between stride length as percentage of body height and ball speed (Table 1; Figure 3A).

The participants had a mean knee angle at FC of 40.3° (SD 14.6, range 10.9–94.6). Linear regression analysis with body height and mound as confounding variables showed that knee angle at FC was not significantly associated with ball speed (Table 1; Figure 3B).

The mean knee angle at MER was 45.0° (SD 17.8, range 9.7–96.0). Body height and mound appeared to bias the association between knee angle at MER and ball speed. After adjusting for these confounders, knee angle at MER was significantly associated with ball speed (Table 1; Figure 3C). The negative regression coefficient found for knee angle at MER indicates that ball speed decreases as the knee angle increase, i.e., as the knee is more flexed. The value of the coefficient (−0.055) shows that youth baseball pitchers who have the knee of their leading leg 1/0.055°, or ~18°, more extended at the moment of MER throw 0.45 m/s (1 mph) faster.

The mean knee angle of the participants at BR was 40.5° (SD 19.0, range 3.9–94.7). The association between knee angle at BR and ball speed appeared to be biased by both body height and mound. After adjusting for these confounders, knee angle at BR was significantly associated with ball speed (Table 1; Figure 3D). Youth baseball pitchers who throw with a 1/0.051°, or ~19.5°, more extended knee at BR pitch 0.45 m/s (1 mph) faster.

## 4. Discussion

The purpose of this study was to determine whether stride length and knee angle of the leading leg at FC, MER and BR were associated with ball speed in elite youth baseball pitchers. In support of our hypotheses knee extension at MER and BR appeared to be significantly and positively associated with higher ball speeds.

The increase in ball speed, which is associated with a more extended knee at MER and BR, is relatively small. To achieve an increase in ball speed of 0.45 m/s (1 mph) a more extended knee of 18–19.5° is required. However, although the effect seems to be small, it still appears statistically significant in the population of the present study, which consists of a homogenous group of youth elite baseball pitchers. The observed result can be an indication of the relevance of knee angle. Moreover, the observed small effect may still have a practical relevance, because at the top level of baseball, small details can make a large difference.

The average stride length in this study was 80% (SD 6) of body height; this is comparable to the stride length found in two other studies [23,24]. However, some other studies found different results [25,26] (Table 2). The latter studies defined stride length as the distance between the centers of the ankle joints and corrected this value for body height, while in the present study the distance between the pitching plate and the center of the ankle joint from the leading leg was defined as the stride length. Since most pitchers place the foot of their trailing leg in front of the pitching plate, the definition of these two studies results in lower relative stride length values. This should be considered for the comparison with the value of this study. The linear regression analysis showed no significant association between ball speed and stride length as percentage of body height in youth baseball pitchers (*β* = 0.022). This association can only be applied to stride lengths within the range of 62–93%, because this study did not measure any stride lengths outside this range. Montgomery & Knudson [17] is the only study that also examined the association between stride length and ball speed and they demonstrated a significant association between the stride length and ball speed. Pitchers in that study had to throw with their normal stride, with under-stride and with over-stride, which was a similar range (75–100%, assuming a body height of 180 cm) as in the present study. However, this study is not comparable with the present study because the association was determined for each pitcher individually, while in the present study associations were determined at group level. It might even be beneficial to have a shorter stride length, since it does not seem to affect ball speed, but it does reduce physical exertion [18]. Overall, more research is needed to understand whether there is an association between stride length and ball speed or not.

The knee angle of the leading leg starts with a mean flexion of 40.3° (SD 14.6) at FC and is followed by more flexion at the time of MER (45.0° SD 17.8). Subsequently, the knee extends towards BR (40.5° SD 19.0), which is consistent with previously published results [26]. However, the knee angle at FC is smaller compared to other studies [23,25] (Table 2). There are two studies which measured the knee angle at MER in youth baseball pitchers [24]. They found a value of 46° (SD 15) and 39° (SD 12.1), which is a result that is comparable to this study (45.0° SD 17.8). The knee angle at BR is within the range of the values found in the other studies [10,23,24]. We found a significant negative association between the knee angle at MER and BR with ball speed. This is similar to the study of Werner et al. [13], which concluded that a higher ball speed (1 mph) is found in pitchers with more knee extension (9°) in the later part of the pitch cycle [10,13]. According to these and our results, youth baseball pitchers should throw with a more extended knee of the leading leg. However, it is important to notice that the present cross-sectional study and the cross-sectional study of Werner et al. [13] only report associations between ball speed and knee angle, which do not support a causal relationship in which a more extended knee would actually lead to higher ball speeds. Therefore, practical implications based on these associations should be critically evaluated. In case of a potential causal relationship, it should be realized that knee extension is limited, which means that the gain in ball speed by more knee extension in the later part of the pitch cycle is limited. It should also be mentioned that at maximal extension, the knee is more vulnerable to injury [27].

The observed association between ball speed and the knee angle of the leading leg might actually be a causal relationship when several mechanical theories are taken into consideration. The extending knee results in a braced leading leg. This results in a braking effect during the stance phase, which means that the leading leg stops moving in a forward direction and the proximal segments rotate over the leading leg. The pitcher should not flex his knee from the moment of FC because this will result in energy dissipation since a non-moving and locked hip (i.e., a fixated trochanter major in space) requires less muscle power of the knee extensors with an extended knee compared to a flexed knee because of the shorter moment arm. The trunk is the segment with the highest mass and is, therefore, potentially one of the greatest force generators in the kinetic chain [4]. Also, in other sports such as javelin throwing, the braking effect of the lead leg is shown to be important because it allows the trunk and upper extremities to accelerate forward over the leading leg, aiding in the transfer of momentum up through the trunk and the throwing arm [28].

In the present study population of youth baseball pitchers, a large range of body types was present due to the obvious effects of growth and maturation at these ages [29,30]. The effect of maturation was, however, not within the scope of the present study. If similar measurements would be performed over time, future studies could focus on the effects of growth in relation to throwing velocity. In the present study, however, we do have to correct for the observed range of body types to arrive at the independent association between the kinematic variables and ball speed. As explained in the methods section, we only used body height as a confounder, as it was expected to largely affect ball speed. Body height itself was highly correlated with body weight, age, and strength (*r* > 0.75) (which were thus all strong predictors of ball speed (*r* > 0.8)). Taking all these confounders into account at the same time in the multiple regression analyses would have introduced issues of collinearity. Therefore, only one of those potential confounding factors needed to be selected for eliminating confounding. Of those factors, body height appeared to be the strongest predictor and was, therefore, the variable chosen for exploring confounding. One should bear in mind that body height should be considered a variable representing other variables such as body weight, age etc., and not only an explanatory variable by itself. In the regression models it was observed that pitchers throw around 0.45 m/s (1 mph) faster for every increase in stature of 0.02 m. Another variable that was explored for confounding, and interaction, was the mound. Pitchers who threw off a mound had a more extended knee at the moments of FC, MER, and BR. Therefore, the mound was included as confounding variable. However, interactions with mound appeared not to be significant. This means that the associations between knee angle and ball speed, and between stride length and ball speed, are not different for the pitchers throwing from a mound and the pitchers not throwing from a mound. Others also reported kinematic differences between pitching off a mound compared to flat ground [31,32,33]. Nissen et al. [31] reported that the knee of the leading leg was in more extension at FC when pitching off the mound. This probably occurs as a consequence of the delay in lead foot contact when stepping down off the mound [32]. Furthermore, ball speed has shown to be different when pitching from flat ground compared to pitching of a mound [33]. This difference in ball speed shows the importance of including the mound as a confounding variable. However, having included pitchers that also do not throw from mound in the present study next to pitcher that do throw from a mound, warrants careful generalization of the results of the present study. In future studies, it should be considered to study the associations between kinematic variables and ball speed preferably when pitchers only throw from a mound, throw only on a flat ground, or do both.

## 5. Conclusions

In conclusion, while stride length and knee angle at FC are not associated with ball speed, more knee extension of the leading leg at MER and BR is associated with higher ball speed in the present sample of elite youth baseball pitchers. Whether pitching with more knee extension at MER and BR can be trained and whether this actually affects the ball speed should be subject of future studies. In addition, future studies should focus on describing the knee angle during the complete pitching cycle and study whether certain characteristics of this time series, for instance the rate of change of the knee angle within a certain phase of the pitch cycle, are associated with ball speed.

## Figures and Tables

**Table 1:** Crude and adjusted associations between lower extremity parameters and ball speed. Confounding variables with their regression coefficient (B) are presented when included in the regression model.

Kinematic Parameters	With or Without Cofounder	B	95% CI	Confounding Variables (B (95% CI))
Relative stride length (%)	Crude	0.046	(−0.082, 0.173)	-
	Adjusted	0.029	(−0.092, 0.150)	Mound (−6.447 (−11.339, −1.555))
Knee angle at FC (degrees)	Crude	0.023	(−0.012, 0.058)	-
	Adjusted	0.031	(−0.002, 0.063)	Body height (cm) (0.476 (0.401, 0.552)), Mound (yes/no) (2.345 (−0.989, 5.680))
Knee angle at MER (degrees)	Crude	−0.058 *	(−0.097, −0.019)	-
	Adjusted	−0.055 *	(−0.088, −0.022)	Body height (cm) (0.459 (0.384, 0.534)), Mound (yes/no) (1.235 (−1.909, 4.379))
Knee angle at BR (degrees)	Crude	−0.053 *	(−0.089, −0.017)	-
	Adjusted	−0.051 *	(−0.083, −0.019)	Body height (cm) (0.466 (0.391, 0.541)), Mound (yes/no) (1.231 (−1.912, 4.374))

(95% CI = 95% confidence interval) * *p* < 0.01.

**Table 2:** Comparison of kinematic parameters between the current study and the literature. For each study, if available, mean (SD) values are presented.

Parameters	Present Study	Milewski et al. (2012)	Fleisig et al. (1999)	Dun et al. (2008)
Age group (years)	15.2 (SD 1.7)	12.4	-	12.5 (SD 1.7)
(Range)	(10.4–18.5)	(10.5–14.7)	(10–15)	(9.8–14.9)
Stride length (% height)	79.8 (SD 6.0)	69 (SD 6)	85 (SD 8)	70 (SD 5)
Knee angle at FC (°)	40.3 (SD 14.6)	49 (SD 12)	43 (SD 12)	49 (SD 8)
Knee angle at MER (°)	45.0 (SD 17.8)	46 (SD 15)	-	-
Knee angle at BR (°)	40.5 (SD 19.0)	41 (SD 16)	36 (SD 11)	31 (SD 9)
